# Diversity of Riparian Plants among and within Species Shapes River Communities

**DOI:** 10.1371/journal.pone.0142362

**Published:** 2015-11-05

**Authors:** Sara L. Jackrel, J. Timothy Wootton

**Affiliations:** Department of Ecology and Evolution, The University of Chicago, Chicago, Illinois, United States of America; Helmholtz Centre for Environmental Research (UFZ), GERMANY

## Abstract

Organismal diversity among and within species may affect ecosystem function with effects transmitting across ecosystem boundaries. Whether recipient communities adjust their composition, in turn, to maximize their function in response to changes in donor composition at these two scales of diversity is unknown. We use small stream communities that rely on riparian subsidies as a model system. We used leaf pack experiments to ask how variation in plants growing beside streams in the Olympic Peninsula of Washington State, USA affects stream communities via leaf subsidies. Leaves from red alder (*Alnus rubra*), vine maple (*Acer cinereus*), bigleaf maple (*Acer macrophyllum*) and western hemlock (*Tsuga heterophylla*) were assembled in leaf packs to contrast low versus high diversity, and deployed in streams to compare local versus non-local leaf sources at the among and within species scales. Leaves from individuals within species decomposed at varying rates; most notably thin leaves decomposed rapidly. Among deciduous species, vine maple decomposed most rapidly, harbored the least algal abundance, and supported the greatest diversity of aquatic invertebrates, while bigleaf maple was at the opposite extreme for these three metrics. Recipient communities decomposed leaves from local species rapidly: leaves from early successional plants decomposed rapidly in stream reaches surrounded by early successional forest and leaves from later successional plants decomposed rapidly adjacent to later successional forest. The species diversity of leaves inconsistently affected decomposition, algal abundance and invertebrate metrics. Intraspecific diversity of leaf packs also did not affect decomposition or invertebrate diversity. However, locally sourced alder leaves decomposed more rapidly and harbored greater levels of algae than leaves sourced from conspecifics growing in other areas on the Olympic Peninsula, but did not harbor greater aquatic invertebrate diversity. In contrast to alder, local intraspecific differences via decomposition, algal or invertebrate metrics were not observed consistently among maples. These results emphasize that biodiversity of riparian subsidies at the within and across species scale have the potential to affect aquatic ecosystems, although there are complex species-specific effects.

## Introduction

Community composition of donor habitats can have large-scale consequences for ecosystem function and community structure in recipient systems. Leaf litter falls from terrestrial plants in riparian zones and provides critical food resources to aquatic invertebrates and microbes residing in small streams. We evaluate how composition of riparian plants at the within and among species scales affects adjacent stream communities and function.

Preservation of biodiversity has been noted as a key means of maintaining ecosystem function [1, 2]. Most studies have focused on species diversity, without considering phenotypic and/or genetic diversity within species. Recent studies have found that population genetic diversity in plants can increase consumer diversity and rates of ecosystem function [3]. Many prior studies have found that rates of leaf litter decomposition in streams are affected by species diversity [2, 4], but few have studied within species variation [5]. Here, we compare the effects of diversity at these two scales, asking whether diversity of terrestrial plants among and within species increases aquatic ecosystem function and effects primary producers and consumers by measuring leaf decomposition, algal abundance, and invertebrate richness and abundance.

Additionally, we ask whether recipient aquatic communities locally adjust to changes in subsidies received from the donor community of riparian plants (both at the among and within species scales) and thereby increase ecosystem function. Leaf litter fuels small stream communities during autumnal leaf senesce, but riparian subsidies are also sizable during summer [6] when food resources are vital for the growing season of many aquatic decomposers [7]. Consequently, aquatic organisms should be efficient consumers of locally available riparian subsidies. Shifting aquatic invertebrate communities with stream size is a well-known example of this local matching: small, narrow stream channels are dominated by leaf-shredding species because these waters receive less sunlight and more leaves per unit of water, while in contrast, the turbid waters of larger streams are instead dominated by collector/filtering species that extract the fine particulate organic matter that is abundant in these channels [8]. Aquatic community matching at finer scales is also possible because the generation time of decomposers is minor relative to the timespan of forest succession and the lifespan of a tree. Indeed, a recent study found that the successional stage of riparian forest affects aquatic decomposition: leaf litter breakdown of certain tree species is higher in stream reaches surrounded by those same riparian tree species [9]. Here, we test whether aquatic decomposer communities locally adjust to leaf litter subsides at these two scales: among tree species (as a comparison with this previous result) and within tree species.

Matching of an aquatic decomposer community could be in any form (such as intraspecific genetic or phenotypically plastic change, or species sorting) that leads to more efficient decomposition of leaf litter. At the interspecific scale, we compare rates of decomposition of red alder (*Alnus rubra*) and western hemlock (*Tsuga heterophylla*) in early versus later successional stretches of river, respectively. We compare our results to a previously documented pattern of more efficient processing of alder leaves in early successional forest in British Columbia [9]. At the intraspecific scale, we compared rates of decomposition of local versus non-locally sourced leaves from three deciduous species (red alder, vine maple and bigleaf maple).

Finally, we test how a well-known driver of intraspecific leaf variation, plant-herbivore interactions, affects aquatic leaf decomposition. We investigated whether plant traits correlated with mechanical and chemical defense to terrestrial herbivory may affect aquatic decomposer communities. We tested whether aquatic decomposition was influenced by the following traits: tree age (because defense often varies with ontogeny [10]), leaf thickness (a frequent means of mechanical defense [11]), and leaf damage due to terrestrial herbivory (as an indicator of past stress and probable induction of a defensive response).

## Materials and Methods

### Ethics Statement

All research was conducted on private lands owned by Merrill and Ring (Pysht Tree Farm). Permission and necessary permits were obtained from Merrill and Ring for the described field studies. No other permits were required. The research involved no endangered or protected species.

### Study Site

We studied aquatic decomposition at 4 sites on the South Fork Pysht River (48.09°N, 124.12°W) in the Olympic Peninsula of Washington State from June–August, 2011. Riparian forest communities varied from early successional forest dominated by red alder to later successional forest dominated by a mix of red alder, bigleaf maple (*Acer macrophyllum*), western hemlock (*Tsuga heterophylla*) and other conifers. We chose four of our river sites to contrast riparian forest communities: we chose sites with similar stream morphology and paired two sites adjacent to early successional forest against two sites adjacent to later successional forest. The early successional sites are along reaches where fire and logging during the 1930's-1970's removed most trees from the area, and alder subsequently recolonized and maintained dominance, resisting invasion of conifers. Later successional sites were intervening river reaches with adjacent pockets of forest in which conifers were not completely removed by harvest and fire, although riparian disturbance and wind throw allowed some alders to establish at these sites too. Previous studies show that reaches throughout this river share similar water chemistry (including SiOH_4_, DOC, PO_4_, NO_3_, NO_2_, NH_4_, N:P), stream composition (% riffle, % pool, water depth variance, % coarse woody debris, % cobble, %silt/sand) and temperature [12].

We added two additional sites on the Pysht River adjacent to early successional forest for our second experimental run. All six sites were between 0.2–3.5 km apart. Common understory vegetation at all sites included salmonberry (*Rubus spectabilis*), vine maple (*Acer cinereus*), thimbleberry (*Rubus parviflorus*), salal (*Gautheria shallon*) and sword fern (*Polystichum munitum*). Additional stream morphology and environmental characteristics of these sites are described elsewhere [6, 12, 13].

### Leaf Pack Experiment

Fresh green leaves are generally more nutrient rich and decompose more rapidly than senescent leaves [14]. In the Pysht River, green leaves fall in large quantities during the summer growing season, decompose quickly, and support high aquatic invertebrate diversity [6]. We hand-picked fresh, green leaves with little or no visible herbivore or pathogen damage from alder, hemlock and maple trees and sealed them in plastic bags. Leaves were collected from the riparian zone of the Pysht river, riparian zones of the Hoko river (26 km away, 48.15°N, 124.21°W) and non-riparian forest in Sekiu (48.15°N, 124.19°W), Clallam Bay (48.16°N, 124.22°W) and Pysht (48.18°N, 124.16°W), WA. We constructed leaf packs containing 12 leaves each using 19 cm x 15.25 cm bags made of 4.75 mm nylon seine netting, this netting size allows colonization by most stream invertebrates (Jackrel, personal observation). We recorded initial weights of leaf packs and deployed packs at each site by attaching them with a cable to a steel reinforcing bar secured on the stream bottom perpendicular to the flow. Seventeen or 18 days after deployment, depending on experimental run, we removed packs and sealed them inside plastic bags. We removed leaves from mesh bags, gently washed them with water to dislodge invertebrates and silt, blotted them dry with paper towels and weighed them to the nearest centigram. We preserved all invertebrates from each leaf pack in 70% ethanol, enumerated and identified all to the lowest possible taxonomic unit using a dissecting scope [15], and calculated diversity using Shannon’s diversity metric [16].

In the first of our two experimental runs, we worked in four riparian sites (two adjacent to early successional forest and two adjacent to later successional forest, as described above) from June 23 –July 10, 2011. We constructed 48 single-individual alder packs (two leaf packs for each of 12 local alder genotypes and each of 12 non-local alder genotypes), 8 mixed-individual alder packs (each containing two leaves from each of six trees: three local and three non-local genotypes), 8 mixed-individual hemlock packs, 8 alder-hemlock mixed-species packs, and 4 alder-maple mixed-species packs (each containing 4 alder, 4 vine maple, and 4 bigleaf leaves). From each individual tree, we constructed duplicate leaf packs and deployed one leaf pack from each duplicate at a site adjacent to early successional forest and the second leaf pack from each duplicate at a site adjacent to later successional forest (Table 1). In the second run from July 25 –August 10, 2011 we asked whether aquatic communities were more efficient decomposers of low versus high species diversity and local versus non-local riparian subsidies at the intraspecific scale in three deciduous plants: red alder, vine maple and bigleaf maple. At the six riparian study sites described above, we deployed 24 single-individual alder packs (two leaf packs for each of six local alders and each of six non-local alders), 24 single-individual vine maple packs, 24 single-individual bigleaf maple packs, and an additional 12 mixed-species packs (each containing 4 alder, 4 vine maple and 4 bigleaf maple leaves) (Table 2).

**Table 1:** Explanation of experimental design used in the first experimental run. Numbers in the contrast columns indicate the contrast coefficients (0, 1, -1, etc.), which were assigned to sum to zero. Bolded and italicized fonts indicate the levels within each contrast comparison as described in the contrast key.

				Contrasts					
Leaf Pack Contents	Treatment Code	Source	Successional Stage of Riparian Zone	1	2	3	4	5	6
Alder Genotype 1	2	Away	Alder (Site 1)	**-1**	**1**	0	0	0	*1*
Alder Genotype 1	4	Away	Conifer (Site 1)	**-1**	**1**	0	0	0	**-1**
Alder Genotype 2	2	Away	Alder (Site 1)	**-1**	**1**	0	0	0	*1*
Alder Genotype 2	4	Away	Conifer (Site 1)	**-1**	**1**	0	0	0	**-1**
Alder Genotype 3	2	Away	Alder (Site 1)	**-1**	**1**	0	0	0	*1*
Alder Genotype 3	4	Away	Conifer (Site 1)	**-1**	**1**	0	0	0	**-1**
Alder Genotype 4	2	Away	Alder (Site 1)	**-1**	**1**	0	0	0	*1*
Alder Genotype 4	4	Away	Conifer (Site 1)	**-1**	**1**	0	0	0	**-1**
Alder Genotype 5	2	Away	Alder (Site 1)	**-1**	**1**	0	0	0	*1*
Alder Genotype 5	4	Away	Conifer (Site 1)	**-1**	**1**	0	0	0	**-1**
Alder Genotype 6	2	Away	Alder (Site 1)	**-1**	**1**	0	0	0	*1*
Alder Genotype 6	4	Away	Conifer (Site 1)	**-1**	**1**	0	0	0	**-1**
Alder Genotype 7	1	Home	Alder (Site 1)	*1*	**1**	0	0	0	*1*
Alder Genotype 7	3	Home	Conifer (Site 1)	*1*	**1**	0	0	0	**-1**
Alder Genotype 8	1	Home	Alder (Site 1)	*1*	**1**	0	0	0	*1*
Alder Genotype 8	3	Home	Conifer (Site 1)	*1*	**1**	0	0	0	**-1**
Alder Genotype 9	1	Home	Alder (Site 1)	*1*	**1**	0	0	0	*1*
Alder Genotype 9	3	Home	Conifer (Site 1)	*1*	**1**	0	0	0	**-1**
Alder Genotype 10	1	Home	Alder (Site 1)	*1*	**1**	0	0	0	*1*
Alder Genotype 10	3	Home	Conifer (Site 1)	*1*	**1**	0	0	0	**-1**
Alder Genotype 11	1	Home	Alder (Site 1)	*1*	**1**	0	0	0	*1*
Alder Genotype 11	3	Home	Conifer (Site 1)	*1*	**1**	0	0	0	**-1**
Alder Genotype 12	1	Home	Alder (Site 1)	*1*	**1**	0	0	0	*1*
Alder Genotype 12	3	Home	Conifer (Site 1)	*1*	**1**	0	0	0	**-1**
Hemlock-Alder Mix 1	9	mixed	Alder (Site 1)	0	0	*-2*	0	*1*	**-1**
Hemlock-Alder Mix 1	10	mixed	Conifer (Site 1)	0	0	*-2*	0	*1*	*1*
Hemlock-Alder Mix 2	9	mixed	Alder (Site 1)	0	0	*-2*	0	*1*	**-1**
Hemlock-Alder Mix 2	10	mixed	Conifer (Site 1)	0	0	*-2*	0	*1*	*1*
Alder Mix 1	5	mixed	Alder (Site 1)	0	*-6*	**1**	*1*	0	*1*
Alder Mix 1	6	mixed	Conifer (Site 1)	0	*-6*	**1**	*1*	0	**-1**
Alder Mix 2	5	mixed	Alder (Site 1)	0	*-6*	**1**	*1*	0	*1*
Alder Mix 2	6	mixed	Conifer (Site 1)	0	*-6*	**1**	*1*	0	**-1**
Hemlock Mix 1	7	mixed	Alder (Site 1)	0	0	**1**	**-1**	0	**-1**
Hemlock Mix 1	8	mixed	Conifer (Site 1)	0	0	**1**	**-1**	0	*1*
Hemlock Mix 2	7	mixed	Alder 1 (Site 1)	0	0	**1**	**-1**	0	**-1**
Hemlock Mix 2	8	mixed	Conifer 1 (Site 1)	0	0	**1**	**-1**	0	*1*
Alder Genotype 13	4	Away	Conifer (Site 2)	**-1**	**1**	0	0	0	**-1**
Alder Genotype 13	2	Away	Alder (Site 2)	**-1**	**1**	0	0	0	*1*
Alder Genotype 14	4	Away	Conifer (Site 2)	**-1**	**1**	0	0	0	**-1**
Alder Genotype 14	2	Away	Alder (Site 2)	**-1**	**1**	0	0	0	*1*
Alder Genotype 15	4	Away	Conifer (Site 2)	**-1**	**1**	0	0	0	**-1**
Alder Genotype 15	2	Away	Alder (Site 2)	**-1**	**1**	0	0	0	*1*
Alder Genotype 16	4	Away	Conifer (Site 2)	**-1**	**1**	0	0	0	**-1**
Alder Genotype 16	2	Away	Alder (Site 2)	**-1**	**1**	0	0	0	*1*
Alder Genotype 17	4	Away	Conifer (Site 2)	**-1**	**1**	0	0	0	**-1**
Alder Genotype 17	2	Away	Alder (Site 2)	**-1**	**1**	0	0	0	*1*
Alder Genotype 18	4	Away	Conifer (Site 2)	**-1**	**1**	0	0	0	**-1**
Alder Genotype 18	2	Away	Alder (Site 2)	**-1**	**1**	0	0	0	*1*
Alder Genotype 19	3	Home	Conifer (Site 2)	*1*	**1**	0	0	0	**-1**
Alder Genotype 19	1	Home	Alder (Site 2)	*1*	**1**	0	0	0	*1*
Alder Genotype 20	3	Home	Conifer (Site 2)	*1*	**1**	0	0	0	**-1**
Alder Genotyp 20	1	Home	Alder (Site 2)	*1*	**1**	0	0	0	*1*
Alder Genotype 21	3	Home	Conifer (Site 2)	*1*	**1**	0	0	0	**-1**
Alder Genotype 21	1	Home	Alder (Site 2)	*1*	**1**	0	0	0	*1*
Alder Genotype 22	3	Home	Conifer (Site 2)	*1*	**1**	0	0	0	**-1**
Alder Genotype 22	1	Home	Alder (Site 2)	*1*	**1**	0	0	0	*1*
Alder Gentotyp 23	3	Home	Conifer (Site 2)	*1*	**1**	0	0	0	**-1**
Alder Genotype 23	1	Home	Alder (Site 2)	*1*	**1**	0	0	0	*1*
Alder Genotype 24	3	Home	Conifer (Site 2)	*1*	**1**	0	0	0	**-1**
Alder Genotype 24	1	Home	Alder (Site 2)	*1*	**1**	0	0	0	*1*
Alder Mix 3	6	mixed	Conifer (Site 2)	0	*-6*	**1**	*1*	0	**-1**
Alder Mix 3	5	mixed	Alder (Site 2)	0	*-6*	**1**	*1*	0	*1*
Alder Mix 4	6	mixed	Conifer (Site 2)	0	*-6*	**1**	*1*	0	**-1**
Alder Mix 4	5	mixed	Alder (Site 2)	0	*-6*	**1**	*1*	0	*1*
Hemlock Mix 3	8	mixed	Conifer (Site 2)	0	0	**1**	**-1**	0	*1*
Hemlock Mix 3	7	mixed	Alder (Site 2)	0	0	**1**	**-1**	0	**-1**
Hemlock Mix 4	8	mixed	Conifer (Site 2)	0	0	**1**	**-1**	0	*1*
Hemlock Mix 4	7	mixed	Alder (Site 2)	0	0	**1**	**-1**	0	**-1**
Hemlock-Alder Mix 3	10	mixed	Conifer (Site 2)	0	0	*-2*	0	*1*	*1*
Hemlock-Alder Mix3	9	mixed	Alder (Site 2)	0	0	*-2*	0	*1*	**-1**
Hemlock-Alder Mix 4	10	mixed	Conifer (Site 2)	0	0	*-2*	0	*1*	*1*
Hemlock-Alder Mix 4	9	mixed	Alder (Site 2)	0	0	*-2*	0	*1*	**-1**
Alder-Maple Mix	12	mixed	Conifer (Site 2)	0	0	0	0	**-4**	**-1**
Alder-Maple Mix	11	mixed	Alder (Site 2)	0	0	0	0	**-4**	*1*
			Sum	0	0	0	0	0	0

Contrast Key: (1) Local matching at within-species scale: leaf packs containing leaves from one *local* vs. **non-locally growing red alder tree**. (2) *High* vs. **low** diversity within species: leaf packs containing 12 leaves from 1 red alder tree vs. 2 leaves from each of 6 red alder trees. (3) *High* vs. **low** species diversity: leaf packs containing leaves from either multiple alder or multiple hemlock vs. mixtures of alder/ hemlock. (4) Contrasting *multiple alder* vs. multiple hemlock mixed individual packs. (5) Contrasting *hemlock/alder* vs. **alder/ vine maple/ bigleaf maple mixed species packs**. (6) Local matching at between species scale: leaf packs contain species that *match* vs. **mismatch** those growing in the adjacent riparian forest.

**Table 2:** Explanation of experimental design used in the second experimental run.

Leaf Pack Contents	Deployment Site	Source
Vine Maple Genotype 1	Site 1	Home
Vine Maple Genotype 1	Site 2	Home
Vine Maple Genotype 2	Site 1	Home
Vine Maple Genotype 2	Site 2	Home
Vine Maple Genotype 3	Site 3	Home
Vine Maple Genotype 3	Site 4	Home
Vine Maple Genotype 4	Site 3	Home
Vine Maple Genotype 4	Site 4	Home
Vine Maple Genotype 5	Site 5	Home
Vine Maple Genotype 5	Site 6	Home
Vine Maple Genotype 6	Site 5	Home
Vine Maple Genotype 6	Site 6	Home
Vine Maple Genotype 7	Site 1	Away
Vine Maple Genotype 7	Site 2	Away
Vine Maple Genotype 8	Site 1	Away
Vine Maple Genotype 8	Site 2	Away
Vine Maple Genotype 9	Site 3	Away
Vine Maple Genotype 9	Site 4	Away
Vine Maple Genotype 10	Site 3	Away
Vine Maple Genotype 10	Site 4	Away
Vine Maple Genotype 11	Site 5	Away
Vine Maple Genotype 11	Site 6	Away
Vine Maple Genotype 12	Site 5	Away
Vine Maple Genotype 12	Site 6	Away
Bigleaf Maple Genotype 1	Site 1	Home
Bigleaf Maple Genotype 1	Site 2	Home
Bigleaf Maple Genotype 2	Site 1	Home
Bigleaf Maple Genotype 2	Site 2	Home
Bigleaf Maple Genotype 3	Site 3	Home
Bigleaf Maple Genotype 3	Site 4	Home
Bigleaf Maple Genotype 4	Site 3	Home
Bigleaf Maple Genotype 4	Site 4	Home
Bigleaf Maple Genotype 5	Site 5	Home
Bigleaf Maple Genotype 5	Site 6	Home
Bigleaf Maple Genotype 6	Site 5	Home
Bigleaf Maple Genotype 6	Site 6	Home
Bigleaf Maple Genotype 7	Site 1	Away
Bigleaf Maple Genotype 7	Site 2	Away
Bigleaf Maple Genotype 8	Site 1	Away
Bigleaf Maple Genotype 8	Site 2	Away
Bigleaf Maple Genotype 9	Site 3	Away
Bigleaf Maple Genotype 9	Site 4	Away
Bigleaf Maple Genotype 10	Site 3	Away
Bigleaf Maple Genotype 10	Site 4	Away
Bigleaf Maple Genotype 11	Site 5	Away
Bigleaf Maple Genotype 11	Site 6	Away
Bigleaf Maple Genotype 12	Site 5	Away
Bigleaf Maple Genotype 12	Site 6	Away
Alder Genotype 1	Site 1	Home
Alder Genotype 1	Site 2	Home
Alder Genotype 2	Site 1	Home
Alder Genotype 2	Site 2	Home
Alder Genotype 3	Site 3	Home
Alder Genotype 3	Site 4	Home
Alder Genotype 4	Site 3	Home
Alder Genotype 4	Site 4	Home
Alder Genotype 5	Site 5	Home
Alder Genotype 5	Site 6	Home
Alder Genotype 6	Site 5	Home
Alder Genotype 6	Site 6	Home
Alder Genotype 7	Site 1	Away
Alder Genotype 7	Site 2	Away
Alder Genotype 8	Site 1	Away
Alder Genotype 8	Site 2	Away
Alder Genotype 9	Site 3	Away
Alder Genotype 9	Site 4	Away
Alder Genotype 10	Site 3	Away
Alder Genotype 10	Site 4	Away
Alder Genotype 11	Site 5	Away
Alder Genotype 11	Site 6	Away
Alder Genotype 12	Site 5	Away
Alder Genotype 12	Site 6	Away
Alder-Maple Mix 1	Site 1	Home
Alder-Maple Mix 1	Site 2	Home
Alder-Maple Mix 2	Site 3	Home
Alder-Maple Mix 2	Site 4	Home
Alder-Maple Mix 3	Site 5	Home
Alder-Maple Mix 3	Site 6	Home
Alder-Maple Mix 4	Site 1	Away
Alder-Maple Mix 4	Site 2	Away
Alder-Maple Mix 5	Site 3	Away
Alder-Maple Mix 5	Site 4	Away
Alder-Maple Mix 6	Site 5	Away
Alder-Maple Mix 6	Site 6	Away

### Chlorophyll Measurements

We asked if leaf pack composition affects algal abundance in the second experimental run. We hypothesized that algal colonization and growth may be affected by leaf traits such as nutrients and/or defense compounds that could leach out of the leaves and that vary within and among species. We have found that alder genotypes vary substantially in leaf Nitrogen and Phosphorus content, and additionally, that the Pysht river may be Nitrogen and Phosphorus co-limited [12, 17]. We quantified algal accrual from each leaf pack by brushing accumulation from each nylon mesh net bag into 100 ml water, homogenizing the sample with a hand blender, and extracting chlorophyll *a* from a 1 ml subsample mixed with 9 ml ethanol in a black vial. Quantity of extracted chlorophyll *a* was measured using a Turner fluorometer with a 440 nm excitation filter and a 665 nm emission filter, calibrated against pure chlorophyll *a* standards derived from spinach (Sigma Aldrich). Note that this procedure measures algae from a standardized surface and area, and that the analysis is targeted toward probing possible effects of leachates from leaves on algae, not toward measuring total algal standing crop living on the surface of fallen leaves, which would be affected by leaf decomposition differences among treatments and by shading from the mesh bags surrounding the leaves.

### Plant Traits

We collected 20 additional leaves per tree in two groups of 10 contiguous leaves starting at the tip of each of two main branches of the tree. We photographed leaves and imported photos into Image J to calculate the fraction of leaf area consumed by terrestrial herbivores. We estimated original leaf area by redrawing the assumed original leaf margin. In cases of very heavy leaf herbivory, we estimated original leaf area using a regression equation relating original leaf area and mid-vein length (original leaf area (cm^2^) = 0.939(mid-vein length in cm)^1.64^; R^2^ = 0.861). We calculated this regression equation from photographs of intact alder leaves (*n* = 276). We then took leaf cores 12 mm in diameter from each of the twenty leaves per tree, oven dried the cores, and weighed each core to the nearest milligram as an indicator of leaf thickness, which often plays an important role in rates of herbivory [11].

### Statistical Analysis

Decomposition rates are reported as percent leaf mass lost over the deployment period (i.e., grams lost over the 17 or 18 days depending on experiment), and data were arcsine-square-root transformed to meet assumptions of normality. For analyses among species, we also measured decomposition as total grams of leaf mass lost. Because the starting weights of leaf packs differed moderately by species due to differences in leaf size (means ± 1 S.D. of alder leaf packs = 17.3g ± 2.15, hemlock leaf packs = 12.9g ± 2.73, vine maple leaf packs = 14.2g ± 2.40, bigleaf maple leaf packs = 22.9g ± 4.08), this measure may more accurately indicate the overall quantity of leaf mass consumed compared to a percentage measure. Except where noted, all analyses using decomposition percentage rate and total grams lost (which are tightly correlated, R^2^ = 0.995) yielded the same biological conclusions. Unless otherwise stated, the unit of replication is the leaf pack. All one-way analysis of variance, multivariate analysis of variance, regression, t-test, and Tukey post-hoc analyses were performed in R (R Development Core Team 2013). The first round of experiments was analyzed with an analysis of variance with six a priori contrast comparisons and was performed in SPSS (IBM Corp. 2013). There were twelve categories of leaf packs for the first experimental round resulting from the combinations of variables, including species identity in a leaf pack, number of species in a leaf pack, the successional stage of the riparian zone adjacent to the deployment location, and the home/away source of the leaves. We provide a detailed explanation of the experimental design and contrast comparisons in Tables 1 and 2. Each of our dependent variables (i.e., leaf decomposition rate, invertebrate diversity, and invertebrate abundance) was analyzed independently using an analysis of variance, and if significant across the twelve treatment groups, the a priori contrast comparisons were analyzed, adjusting contrast coefficients as necessary to maintain balance among treatments when estimating group means (as shown in Tables 1 and 2). For the second round of experiments, we performed one-way analysis of variance tests without predefined contrasts (this experimental design is explained in detail in Table 2). Where indicated, data presented in figures were controlled statistically for deployment location by using residuals to standardize results across multiple sites.

## Results

### Effects of interspecific leaf variation

#### Differences between tree species

We saw strong species effects on decomposition: in our first experiment mixed-individual alder packs decomposed proportionately more rapidly than mixed-individual hemlock packs (F_1,73_ = 4.10, p = 0.047, Tables 1 and 3) although the difference in total mass loss was statistically weaker (p = 0.09). Leaves showed extensive skeletonization and distinct bite marks by invertebrates, but our metrics of invertebrate abundance and richness explained few of the differences we observed in decomposition rates. Leaf packs across treatment groups, including this mixed-alder versus mixed-hemlock comparison, were colonized by a similar abundance (F_11,73_ = 1.1, p = 0.39) and diversity (F_11,73_ = 0.56, p = 0.86) of stream invertebrates. Additionally, all treatment groups were colonized by similar numbers of families belonging to different functional feeding groups (including: shredders, scrapers, predators, collector-gathers, and collector-filterers, MANOVA: F_11,73_ = 0.95, p = 0.58), however the abundance of individuals belonging to each of these functional groups differed across treatment groups (MANOVA: F_11,73_ = 1.68, p < 0.01). Specifically, predators, but not other functional feeding groups, differed across treatments (F_11,73_ = 3.8, p < 0.001, Table 4): there was a significant species effect, where predators were more abundant among mixed-alder packs than hemlock packs (F_1,73_ = 5.44, p = 0.022).

**Table 3:** Summary of leaf decomposition results from first experimental run of leaf packs.

	df	SS	MS	F	P
Leaf pack Treatments	11	1.203	0.109	6.55	< 0.0001
Within-species matching^1^	1	0.707	0.707	42.38	< 0.0001
Within-species diversity^2^	1	0.032	0.032	1.94	0.168
Species diversity^3^	1	0.041	0.041	2.41	0.122
Species comparison^4^	1	0.068	0.068	4.10	0.047
Mixed species comparison^5^	1	0.096	0.096	5.74	0.020
Species matching^6^	1	0.130	0.130	7.81	0.007
Error	62	1.035	0.017		
Total	73	2.238			

A priori contrasts:

^(1)^ Local matching at within-species scale: leaf packs containing leaves from one local vs. non-locally growing red alder tree;

^(2)^ High vs. low diversity within species: leaf packs containing 12 leaves from 1 red alder tree vs. 2 leaves from each of 6 red alder trees;

^(3)^ High vs. low species diversity: leaf packs containing leaves from either multiple alder or multiple hemlock vs. mixtures of alder/ hemlock;

^(4)^ contrasting multiple alder vs. multiple hemlock mixed individual packs

^(5)^ contrasting hemlock/alder vs. alder/ vine maple/ bigleaf maple mixed species packs;

^(6)^ local matching at between species scale: leaf packs contain species that match vs. mismatch those growing in the adjacent riparian forest.

**Table 4:** Abundance of predatory aquatic invertebrates among leaf pack treatments from first experimental run.

	df	SS	MS	F	P
Leaf pack Treatments	11	156.2	14.2	3.81	< 0.001
Within-species matching^1^	1	6.02	6.02	1.62	0.21
Within-species diversity^2^	1	0.03	0.03	0.01	0.92
Species diversity^3^	1	0.33	0.33	0.09	0.77
Species comparison^4^	1	20.25	20.25	5.44	0.022
Mixed species comparison^5^	1	67.6	67.6	18.2	< 0.001
Species matching^6^	1	1.64	1.64	2.27	0.14
Error	62	230.9	3.724		
Total	73	387.1			

The a priori contrasts are the same as those reported in Table 3:

^(1)^ Local matching at within-species scale: leaf packs containing leaves from one local vs. non-locally growing red alder tree;

^(2)^ High vs. low diversity within species: leaf packs containing 12 leaves from 1 red alder tree vs. 2 leaves from each of 6 red alder trees;

^(3)^ High vs. low species diversity: leaf packs containing leaves from either multiple alder or multiple hemlock vs. mixtures of alder/ hemlock;

^(4)^ contrasting multiple alder vs. multiple hemlock mixed individual packs

^(5)^ contrasting hemlock/alder vs. alder/ vine maple/ bigleaf maple mixed species packs;

^(6)^ local matching at between species scale: leaf packs contain species that match vs. mismatch those growing in the adjacent riparian forest.

**Fig 1:**
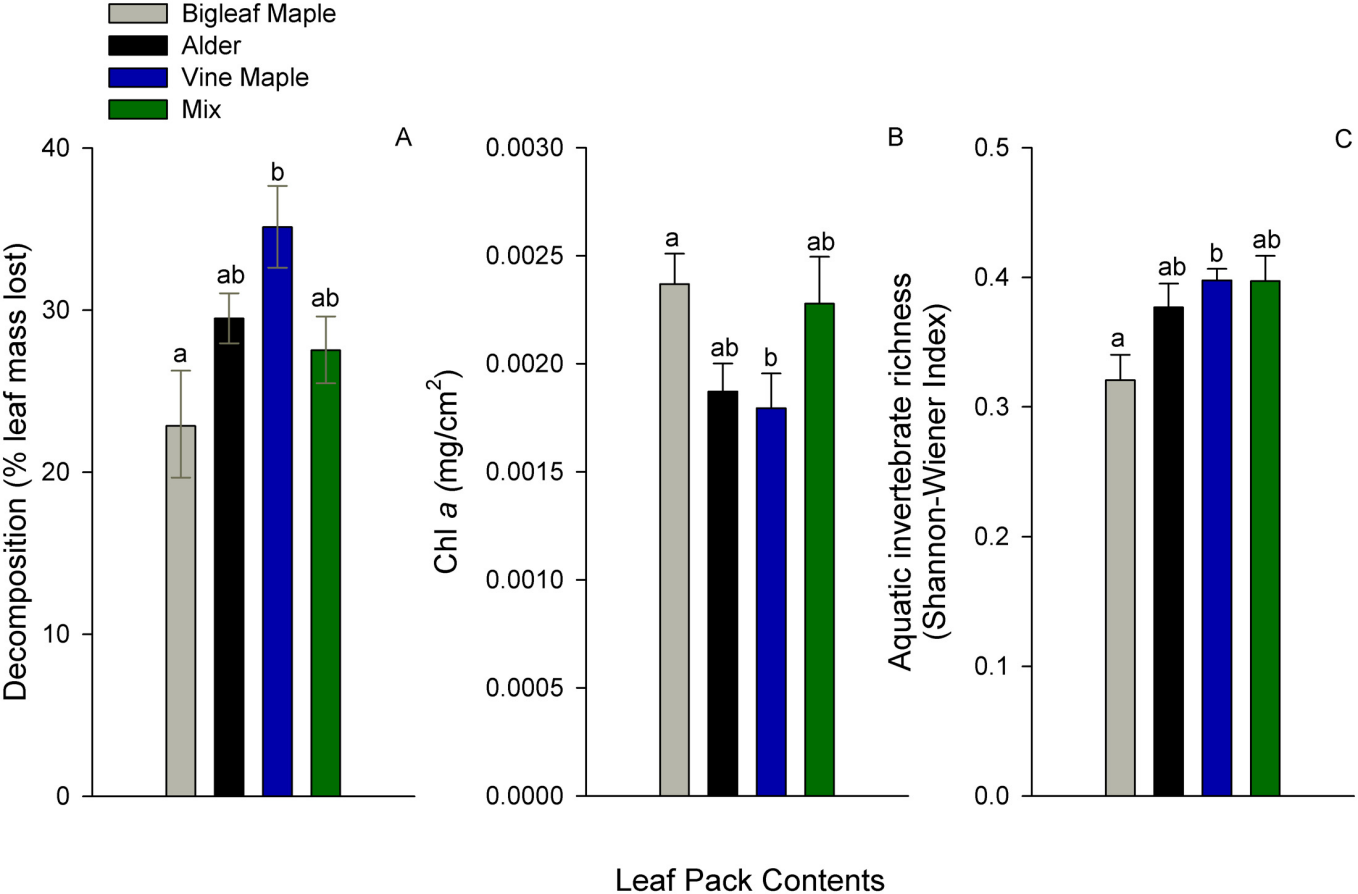
Biological responses to among species variation of leaf packs (mean ± 1 se). (A) Decomposition rates, (B) algal accrual and (C) aquatic invertebrate richness (Shannon’s diversity index) of alder (n = 24), vine maple (n = 24) and bigleaf maple (n = 24) in single and mixed species leaf packs (n = 12). Significant comparisons (P < 0.05) indicated by a star.

In the second experiment, bigleaf maple proportionately decomposed most slowly and vine maple decomposed most rapidly (one-way ANOVA: F_3,38_ = 4.04, p = 0.014, Tukey HSD: vine maple/bigleaf maple comparison, p < 0.01), although the total amount of weight loss was not different between species, owing to the larger leaves of bigleaf maple (Tukey HSD for the vine maple/bigleaf maple comparison, p = 0.108) (Fig 1a). Aligned with this pattern, bigleaf maple supported lower invertebrate diversity than vine maple (one-way ANOVA: F_3,38_ = 3.95, p = 0.015, Tukey HSD: vine maple/bigleaf maple comparison, p = 0.017) (Fig 1c), but harbored greater algal accrual than vine maple (one-way ANOVA: F_3,38_ = 3.56, p = 0.023, Tukey HSD: p = 0.039) and slightly greater algal accrual than alder (Tukey HSD: p = 0.090).

#### Effects of diversity among tree species

In the first experiment, multi-species leaf packs (mixed alder/hemlock) and single-species leaf packs (mixed individuals of alder or mixed hemlock) decomposed at similar rates (F_1,73_ = 2.41, p = 0.12). However, among multi-species packs, alder/vine maple/bigleaf maple mixed-species packs tended to decompose more rapidly than alder/hemlock mixed packs (F_1,73_ = 5.74, p = 0.020, Table 3), and these mixed-species deciduous packs also harbored a greater abundance of invertebrate predators than alder/hemlock packs deployed at the same time (F_1,73_ = 18.2, p < 0.001, Table 4).

These diversity comparisons could have been affected by proportional differences in mass contributions of alder versus hemlock to the mixed-species leaf packs, given that decomposition rates varied among species. Therefore, we completed a more targeted comparison of high versus low species diversity using decomposition rates measured in both single and mixed-species leaf packs. We calculated expected decomposition rates for mixed alder-hemlock packs using decomposition data for mixed-individual alder packs and mixed-individual hemlock packs that were deployed at the same location and time as the mixed-species packs. We weighted averages by the starting biomass composition of alder and hemlock in each mixed-species pack. Mixed-species packs lost twice as much leaf mass as expected (paired t-test of expected vs. observed decomposition rate, t_7_ = 3.4, p = 0.011), but expected and observed invertebrate diversity and abundance were similar (paired t-tests, p > 0.1).

In the second experimental run, we observed similar decomposition rates among high and low species diversity packs (either compared to each species individually: alder, vine, or bigleaf maple, all Tukey HSD p > 0.01, or as a group: F_1,40_ = 0.12, p = 0.73), but in contrast to the first experiment, we found invertebrate abundances were lower among single-species (alder, vine maple or bigleaf maple) compared to mixed-species packs (enumeration F_1,40_ = 5.106, p = 0.030). The diversity of invertebrates did not differ between low and high tree-species diversity packs (Shannon diversity F_1,40_ = 1.131, p = 0.29, Fig 1c). Algal accrual was also similar between high tree-species diversity packs and single tree species packs (either compared to each species individually: alder, vine or bigleaf maple, all Tukey HSD p > 0.1, or as a group: F_1,40_ = 1.218, p = 0.28) (Fig 1b).

We also did a more targeted diversity comparison of single-species versus alder-maple packs. We estimated starting composition of each species in the mixed-species packs using average weight data from single-species packs. Expected decomposition rates were calculated using data from single-species packs that were deployed at the same location and time as the mixed alder-maple packs. In contrast to the first round, we found no evidence of accelerated decomposition in high diversity packs (paired t-test t_11_ = 0.34, p = 0.77). Observed and expected values of algal abundance, invertebrate diversity and invertebrate abundance were all similar (paired t-tests, all p > 0.1).

### Effects of intraspecific leaf variation

#### Individual Differences

In the first and second experiments, we found that different aquatic communities responded consistently to different alder genotypes: 24 replicate leaf packs constructed from 12 trees and deployed at two different sites exhibited highly correlated decomposition rates (R^2^ = 0.75, p < 0.001). Bigleaf maple, but not vine maple, also showed consistent results across sites for a given individual (BLM: R^2^ = 0.57, p < 0.01, *n* = 12; VM: R^2^ = 0.19, p = 0.16, *n* = 12). Although genotypes showed varying decomposition rates, this variation within species was not correlated with either algal abundance (second experiment) or invertebrate abundance or diversity (either experiment) (alder, bigleaf maple and vine maple all p > 0.05).

#### Plant Traits Affecting Individual Differences

**Fig 2:**
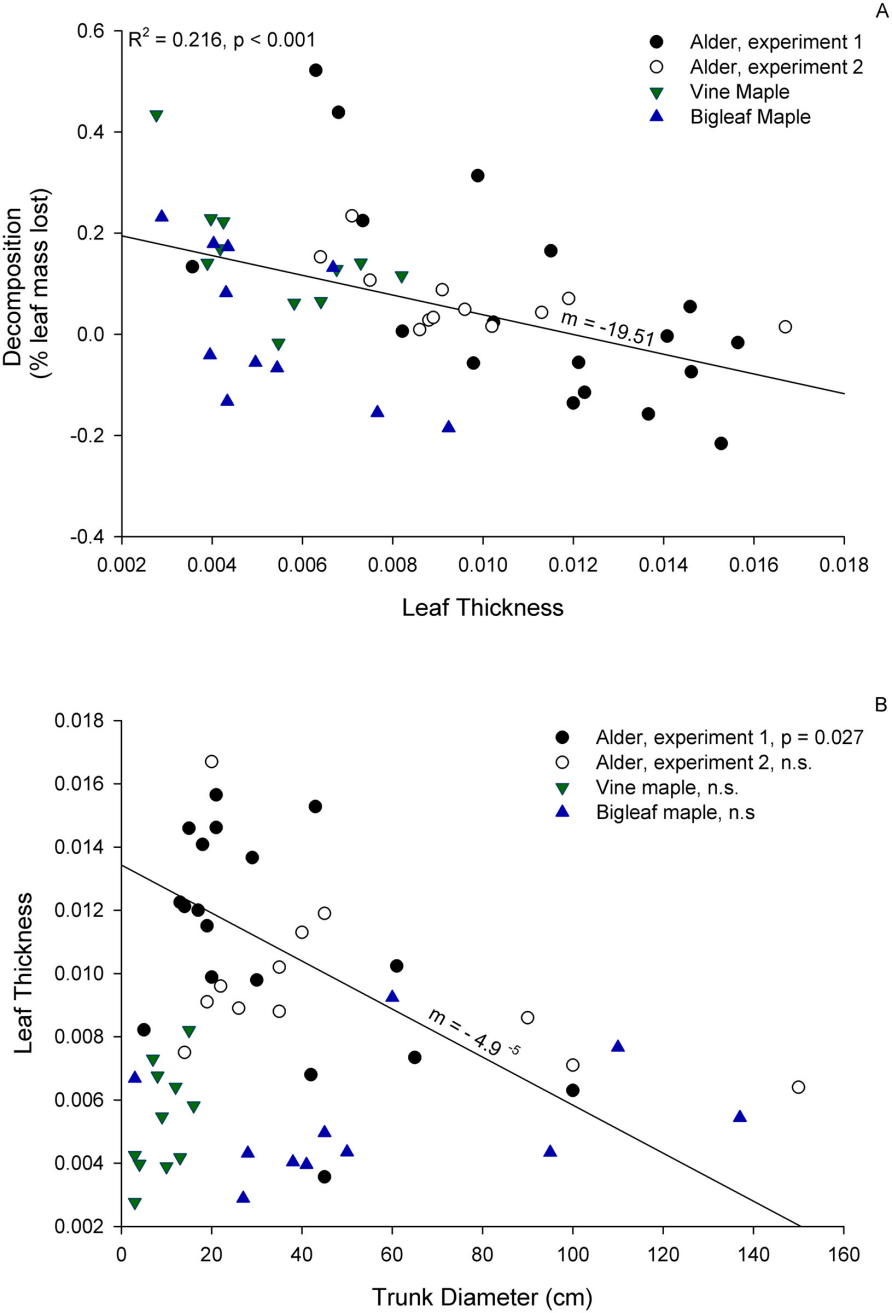
Plant traits influencing the degree of aquatic decomposition of deciduous leaves over the experimental period. (A) Correlation between decomposition and leaf thickness are shown for each species independently and combined (note negative decomposition rates indicate mass gained), and (B) correlation between trees size and leaf thickness are shown for each species independently to illustrate species differences.

Alder, vine maple and bigleaf maple with thinner leaves tended to show greater decomposition (either as a Pearson correlation between decomposition and leaf thickness, R^2^ = 0.216, p < 0.001, or as a model term of decomposition as a function of leaf thickness (F_1,36_ = 7.46, p < 0.01), tree diameter and species) (Fig 2a). There was no common relationship across species between tree diameter and leaf thickness: larger alder trees tended to have thinner leaves (alder experiment 1: R^2^ = 0.27, p = 0.027; alder experiment 2: R^2^ = 0.24, p = 0.10), larger vine maples tended to have thicker leaves (R^2^ = 0.278, p = 0.095) and bigleaf maple showed no pattern (R^2^ = 0.00, p = 0.96) (Fig 2b). Possibly as a consequence of this inconsistent relationship between tree diameter and leaf thickness, we found trunk diameter, as a proxy of age, was a significant but inconsistent predictor of decomposition rate (F_1,36_ = 21.88, p < 0.001; tree diameter*species interaction = F_1,36_ = 2.56 p = 0.07). Leaves from larger alder tended to decompose more rapidly than smaller alder, while leaves from smaller vine maple decomposed more rapidly than larger vine maples (alder: R^2^ = 0.177, p = 0.021; maple: R^2^ = 0.504, p = 0.015). Smaller individuals of bigleaf maple also decomposed more rapidly than larger individuals(R^2^ = 0.425, p = 0.030), however given the lack of relationship between tree diameter and leaf thickness, this result seems to be driven by some other ontogenetic factor.

Terrestrial herbivory was not correlated with alder decomposition rate in either run of experiments (experiment 1, *n* = 18, R^2^ = 0.006, p = 0.76; experiment 2, *n* = 12, R^2^ = 0.011, p = 0.74). We did not estimate terrestrial herbivory for maples.

#### Intraspecific Diversity

In the first experiment, we found that despite alder genotypes showing a range of decomposition rates, single and multi-individual alder leaf packs did not exhibit significantly different decomposition rates (F_1,73_ = 1.94, p = 0.17, Table 3). Single versus mixed-individual alder packs also harbored similar invertebrate abundance (F_1,26_ = 1.801, p = 0.19) and diversity (F_1,26_ = 0.517, p = 0.48). Additionally, we calculated expected decomposition rates and invertebrate metrics for each mixed-individual alder pack using data from single-individual alder packs deployed at the same deployment location and time as the mixed packs. We found no evidence of accelerated decomposition in mixed-individual alder packs (paired t-test: t_7_ = 0.99, p = 0.36), and observed versus expected values for invertebrate diversity and abundance were similar (paired t-tests, p > 0.1)

### Recipient community matching to donor subsidies

#### Species Scale

**Fig 3:**
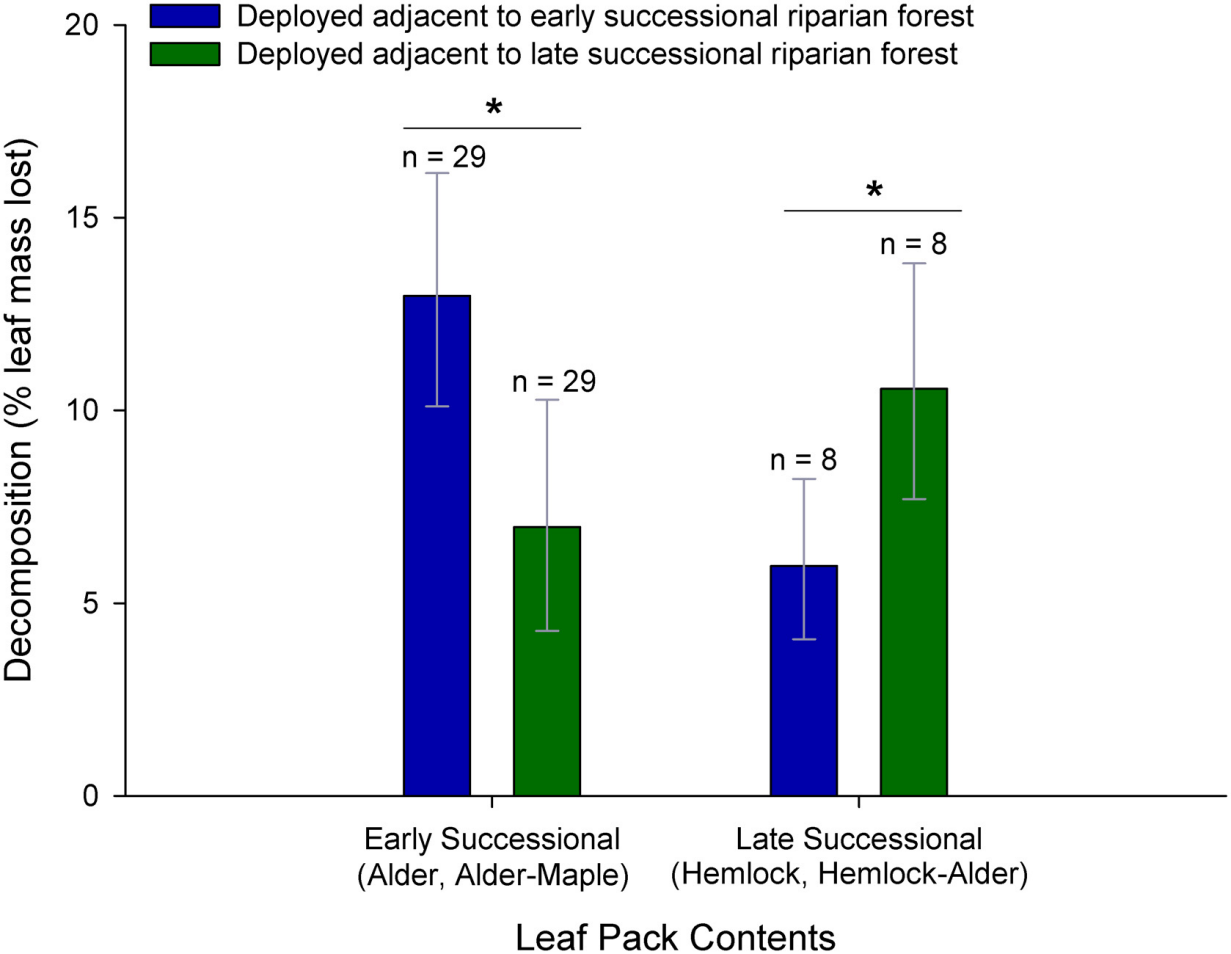
Leaf decomposition rate when leaf contents match the successional stage of adjacent riparian forest. Comparative decomposition (mean ± 1 se) of leaf packs containing early (alder or a mix of alder, vine maple and bigleaf maple) versus later successional species (hemlock or a mix of hemlock and alder) deployed at sites adjacent to early versus later successional riparian forest. Significant comparisons (paired t-tests P < 0.05) indicated by a star.

Aquatic communities more efficiently processed leaf litter when the species composition matched the local successional stage of the adjacent forest: leaf packs including both local and non-local genotypes for each species decomposed more rapidly when deployed at sites surrounded by riparian forest of matching composition to that of the pack contents (F_1,73_ = 7.82, p = 0.007, Table 3). Specifically, alder packs and alder-maple packs decomposed more rapidly at sites surrounded by early successional forests while hemlock packs and alder-hemlock packs exhibited the opposite trend of more rapid decomposition at sites surrounded by later successional forest (Fig 3).

#### Within Species Scale

**Fig 4:**
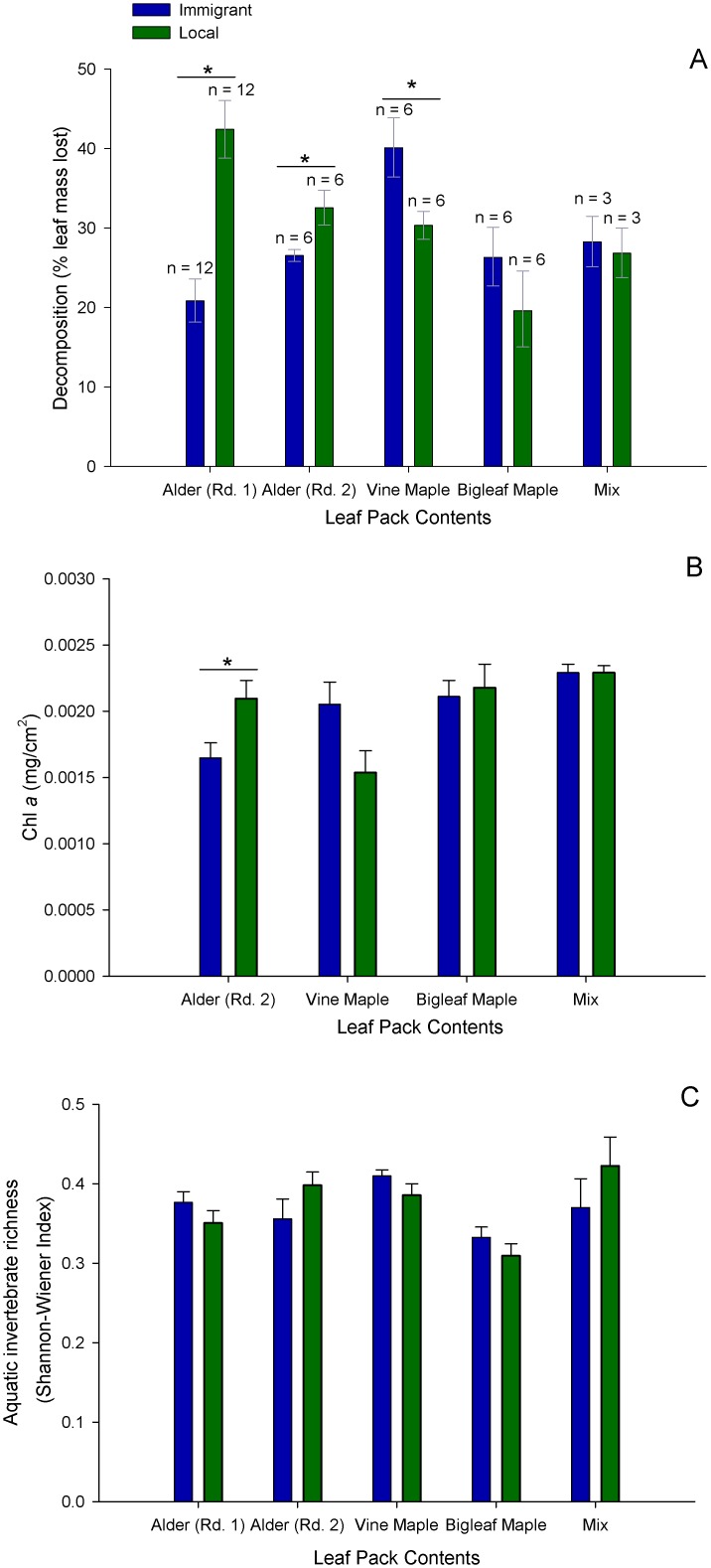
Biological responses to variation at the within-species scale (mean ± 1 se). (A) decomposition rates, (B) algal accrual and (C) aquatic invertebrate Shannon diversity richness of local versus immigrant alder (from two experimental runs), vine maple, bigleaf maple and mix packs containing all three species. Significant comparisons (P < 0.05) indicated by a star.

This local preference pattern was also found at the intraspecific scale, but only for alder, the most abundant deciduous tree. Alder leaves sourced from local trees growing along the banks of the Pysht river decomposed significantly more than immigrant alder leaves (Run 1: F_1,73_ = 42.38, p < 0.001, Table 3; Run 2: F_1,10_ = 7.08, p = 0.024). Interestingly, local alder packs that generally showed accelerated decomposition rates also harbored greater algal abundances than immigrant packs (alder: F_1,10_ = 6.25, p = 0.031) (Fig 4b). Despite rapid decomposition of local alder leaves, neither invertebrate abundance nor diversity differed between local and immigrant leaf packs of alder (p > 0.1, Fig 4c).

Leaves from immigrant vine maple tended to decompose more quickly than leaves from local vine maple (F_1,10_ = 5.77, p = 0.037). Local versus immigrant packs showed similar rates of decomposition for bigleaf maple (F_1,10_ = 1.18, p = 0.3) and mixed-species packs (F_1,4_ = 0.10, p = 0.76). Matching the similar decomposition rates we observed for these comparisons, algal accrual on local and immigrant packs of vine maple, bigleaf maple, and mixed packs was similar (all p > 0.05). There were also no differences in invertebrate abundance or diversity between local and immigrant vine maple, bigleaf maple or mixed-species packs (all p > 0.1, Fig 4c). These invertebrate results statistically control for deployment site because we found general differences in the aquatic communities inhabiting early versus later successional sites: aquatic invertebrate diversity was greater on leaf packs adjacent to later successional riparian forest than early successional forest (paired t-tests (means ± se): later 0.43 ± 0.022 se, early 0.37 ± 0.017, t_13_ = 2.22, p = 0.045), however abundance was greater on leaf packs adjacent to early successional riparian forest (early riparian 46 ± 4.6 se individuals, later riparian 74 ± 9.0, t_13_ = 2.89, p = 0.013).

Local, riparian alder tended to have thinner leaves than immigrant alders growing in non-riparian forests (F_1,16_ = 17.2, p < 0.001). Therefore in our second experiment we included several riparian alder collected along the Hoko River, some of which had leaves as thin as alder on the Pysht. Although the immigrant group as a whole had slightly thicker leaves, F_1,10_ = 5.67, p = 0.039, when comparing six immigrant and local individuals with equally thin leaves (t_3_ = 0.58, p = 0.59), immigrant leaves still tended to decompose less (t_3_ = 3.54, p = 0.023).

## Discussion

### Effects of interspecific leaf variation

Species ordering of decomposition rates (most rapid: vine maple, alder, bigleaf maple: least rapid) in the Pysht river appears to be general [18]. Aligned with this pattern of interspecific decomposition, vine maple tended to support higher invertebrate diversity than bigleaf maple. We found species diversity had inconsistent effects on decomposition. Alder-hemlock packs decomposed substantially more than expected based on decomposition rates of either species alone. In contrast, we found high diversity packs of deciduous species decomposed additively, and there was no significant increase in invertebrate diversity with increasing interspecific tree diversity. This lack of synergistic decomposition among the mixed deciduous species packs matches some previous studies [19], but is in contrast to literature reviews that find non-additive effects in up to 80% of studies [20, 21]. A possible explanation for why diversity did not affect decomposition or invertebrates is that while our mixed species packs increased richness, the larger size of bigleaf maple leaves likely led to lower evenness than intended.

### Effects of intraspecific leaf variation

At the intraspecific scale, we found substantial individual variation in decomposition rate of alder leaves, but leaf packs with high intraspecific diversity decomposed additively compared to single-individual packs. There was also no significant change in invertebrate diversity with increasing intraspecific tree diversity. We tested whether intraspecific variation was due to varying degrees of terrestrial herbivory damage, but we found that our estimates of terrestrial herbivory did not scale with aquatic decomposition. We hypothesized that herbivores and decomposers may have similar feeding preferences if both favor high nutrient quality. Alternatively, herbivore preferences could be negatively correlated with decomposer preferences if feeding by herbivores induces plant defenses that deter decomposers. To detect whether terrestrial herbivory indirectly affects aquatic decomposition, more directed experiments are necessary to untangle how constitutive and induced properties of leaves drive feeding preferences. Previous studies have demonstrated mammalian browsing [22] and insect defoliation [23] are positively associated with rapid stream decomposition of leaves, however, these studies did not disentangle correlation from causation. In a more targeted study of experimentally inducing defenses in isolation and in tandem with nutrient additions to red alder trees [17], we show that a red alder tree’s response to herbivory stress does in fact strongly deter aquatic decomposition of leaf litter. In our previous study, we found this reduction in decomposition was due in part to a sharp reduction in leaf nitrogen content that was a consequence of the herbivory stress[17].

### Recipient community matching to donor subsidies

We found evidence of recipient aquatic communities matching to donor subsidies from riparian forest at the species and within-species scales. Aquatic decomposers more efficiently processed leaf litter that matched the successional stage of immediate riparian forest: alder decomposed more rapidly in stream reaches surrounded by alder dominated forest and hemlock decomposed more rapidly adjacent to later successional forest. This result is similar to a previous study by Kominoski et al., which found alder decomposed more quickly adjacent to deciduous forest [9]. In our study we found a reciprocal pattern with hemlock, while Kominoski et al. found hemlock decomposed independently of forest composition. Whether this discrepancy arises from sample size differences or from geographic differences in leaves and decomposers requires further study. One possible explanation for this difference would be if the British Columbia study sites experienced less severe disturbance (such as logging or scouring floods) than the Washington sites, and consequently have had a more persistent conifer forest and therefore stronger selective pressure to retain efficiency for decomposing hemlock.

Leaves from local alder trees along the Pysht River decomposed more than alder leaves from trees growing in more distant areas. Studies of *Alnus glutinosa* have found intraspecific variation in stream decomposition at a continental scale [5], however our result suggests local population differences among trees within a single region. Generally individuals with thinner leaves tended to have faster leaf decomposition rates, and while we cannot exclude the possibility that differences in leaf thinness may have contributed to the difference between local and immigrant alder leaves in our first experimental run, alder leaves collected from the riparian zone of a distant river often had leaves as thin as local leaves but still decomposed less.

This result spurred a targeted study of local matching of aquatic communities to intraspecific variation in alder leaves [6], in which we showed, using reciprocal transplant experiments across four rivers, that aquatic communities strongly prefer local alder leaves at both the larger across-river scale and the within-river scale. Our experiments in these rivers suggest that aquatic decomposer communities are locally matched to intraspecific variation in riparian subsidies. However, we found no evidence of local matching to variation in either vine maple or bigleaf maple. This inconsistency might be explained by tradeoffs in the aquatic community to efficiently handling different local plant species. Aquatic communities seem to most efficiently process alder, the most abundant deciduous tree, which provides as much as 95–98% of the total summer leaf fall in rivers in the Olympic Peninsula [3], at the expense of efficiently processing leaves of less common species. Additionally, although local alder leaf packs decomposed significantly more than immigrant leaf packs, we saw similar invertebrate communities colonize the two types of leaf packs.

### Conclusions

Overall, our metrics of invertebrate abundance and richness explained few of the differences we observed in decomposition rates. Invertebrate communities may have caused a large portion of the variation in leaf decomposition rates observed, but our single sample of the invertebrate community per leaf pack may have been insufficient to detect differences among invertebrate communities. Alternatively, either within-species differences in invertebrates or shifts in microbial decomposer communities may be playing a large role in differentially decomposing leaf packs.

Our algal abundance measures were intended to determine whether leachates from leaves that vary among and within species of trees have implications for adjacent algal communities. Algal abundance did not scale with intraspecific variation in decomposition. Our interspecific results showed bigleaf maple packs harbored the greatest algal abundance and the lowest decomposition rates. In contrast, local alder leaf packs decomposed faster but harbored greater algal abundance than immigrant alder packs. This reversed pattern might be explained by differences in interspecific leaf chemistry affecting decomposition. Elsewhere we show that rapidly-decomposing alder leaves contain high nitrogen concentrations [17], which might stimulate surrounding algal growth when leached from the leaves. If reduced decomposition in bigleaf maples instead arises from production of defensive compounds, their release into the surrounding water may offer a local algal refuge by repelling algal grazers.

Our results document how biodiversity among and within species can have cascading effects on aquatic organisms receiving significant subsidies. Although we did not measure strong effects of diversity at the leaf pack scale, our finding of local matching suggests biodiversity is a critical component shaping ecosystem functioning. Diversity across reaches evidently facilitates adaptive processes that increase decomposition efficiency at two scales: aquatic communities showed local matching to early versus later successional stages of riparian forest, as well as local matching to within species differences in alder genotypes. This biological variation in plant traits may be the consequence of innumerable terrestrial processes, such as terrestrial herbivory, nutrient and water availability, and frequency and intensity of natural and anthropogenic disturbance. Understanding how these different terrestrial processes shape plant traits will be important in predicting consequences of environmental changes on recipient systems. The effects of diversity from donor communities versus authochtonous diversity within the recipient system may be a worthwhile future comparison due to differences in feedbacks that may alter selection pressures for efficiency. Maintaining maximal ecosystem function may depend on maintaining local diversity at a scale often overlooked in conservation and restoration efforts. Determining the temporal scale of these processes is essential for predicting how rapid anthropogenic changes may disrupt across-ecosystem linkages. To determine the relevant spatial scale and why local preferences are evident in some cases but not others, we need further studies to unravel the mechanisms underlying these apparently adaptive responses of ecological communities.
